# Interplay Between Periodontal Health and Renal Function in Terms of Severity of Chronic Kidney Disease: A Comprehensive Review

**DOI:** 10.7759/cureus.110190

**Published:** 2026-06-03

**Authors:** Krish Relan, Ankrishik Sardar, Katherine Rodriguez, Daniel Leyva, Mary Girgis, Jinal Choudhari, Sultan Ahmed, Echols Marti, Komal Relan

**Affiliations:** 1 Medicine, University of Miami, Coral Gables, USA; 2 Biology, Nova Southeastern University, Davie, USA; 3 Medicine, Ponce Health Sciences University, Ponce, PRI; 4 Biology, Florida International University, Miami, USA; 5 Medicine, University of Florida, Gainesville, USA; 6 Medicine, Larkin Community Hospital, South Miami, USA; 7 Research and Academic Affairs, Larkin Community Hospital, Miami, USA; 8 Dentistry, Bahri Dental Group, Jacksonville, USA

**Keywords:** ckd, dental hygiene, esrd, gingivitis, gum disease, periodontal disease, periodontitis, renal function

## Abstract

Chronic kidney disease (CKD) and periodontal disease (PD) represent significant global health burdens with an increasingly recognized, yet mechanistically complex, interrelationship. This review examines current literature from 2015 to 2025 on the association between dental health and renal function, with select foundational studies from 2008 to 2014 retained for mechanistic context and explicitly disclosed as such. We explored shared biological mechanisms, including systemic inflammation, oxidative stress, impaired neutrophil function, and altered vitamin D metabolism, and identified a critical research gap regarding the definitive direction of causality. By synthesizing clinical data on biomarkers and management challenges, this review provides a direction for future longitudinal research aimed at clarifying the periodontal disease-CKD axis and supports the adoption of integrated dental-nephrological care models.

## Introduction and background

Periodontal disease (PD) is a chronic, multifactorial inflammatory disease associated with a dysbiotic oral microbiome, leading to progressive destruction of the tooth-supporting periodontium [1]. Chronic kidney disease (CKD) is characterized by a glomerular filtration rate (GFR), a measure of the kidneys' blood-filtering efficiency, below 60 mL/min/1.73m² or other markers of kidney damage persisting for more than three months [2].

Recent epidemiological evidence highlights a bidirectional relationship between CKD and PD [3]. Compromised periodontal health can accelerate renal decline, likely via sustained bacteremia and systemic inflammation, while progressive renal failure can, in turn, worsen oral pathology through uremia, immune suppression, and altered mineral metabolism. Thus, a self-reinforcing cycle with measurable clinical consequences emerges. Supporting this, epidemiological data show patients with moderate-to-severe PD have a 2.5-fold increased prevalence of CKD and nearly double the risk of renal decline compared to periodontally healthy controls [4]. Conversely, the uremic environment of CKD often exacerbates oral pathology, causing further deterioration of renal and periodontal health [5]. This systemic interplay, mediated by shared inflammatory pathways and oxidative stress, underscores the need for a multidisciplinary approach to management [6].

Given that both conditions disproportionately burden populations with diabetes mellitus (DM) and cardiovascular disease, clarifying their interaction carries direct implications for preventive care and multidisciplinary management. Building on the clinical and mechanistic insights described above, this review synthesizes current evidence (2015-2025) on the epidemiology, shared biological mechanisms, clinical biomarkers, management challenges, and future research priorities of the PD-CKD axis.

## Review

Methods

Search Strategy

A systematic literature search was conducted across PubMed, PubMed Central, and Google Scholar. The following search terms were used: ("Chronic Kidney Disease," "CKD," "renal failure," or "renal insufficiency") and ("Periodontal Disease," "periodontitis," "dental hygiene," or "gingival inflammation"). Boolean operators AND and OR combined and expanded these terms. The search included publications from January 2015 to December 2025. A narrative synthesis approach was used, as the study design, since periodontal case definitions and renal biomarker reporting varied greatly. This heterogeneity prevented formal statistical pooling.

Inclusion and Exclusion Criteria

Studies were included if they met all of the following criteria: (1) Patient age ≥ 18 years; (2) Human subjects only; (3) Published between January 2015 and December 2025; (4) Study designs including randomized controlled trials (RCTs), clinical studies, case reports, review articles, and phase three or four clinical trials.

Studies were excluded if they involved: (1) Patients younger than 18 years; (2) In vitro studies; (3) Animal studies; (4) Kidney transplant patients; (5) Patients with other chronic inflammatory conditions, to minimize confounding variables

Select studies published prior to January 2015 were retained where they provided foundational mechanistic evidence unavailable in more recent literature (e.g., neutrophil dysfunction pathways, glomerular filtration rate (GFR) classification criteria). These pre-2015 references are explicitly identified as foundational studies.

This manuscript does not carry a pre-registered protocol on the International Prospective Register of Systematic Reviews (PROSPERO), and this limitation is acknowledged.

Study Selection and the Preferred Reporting Items for Systematic Reviews and Meta-Analyses (PRISMA) Flow

**Figure 1:**
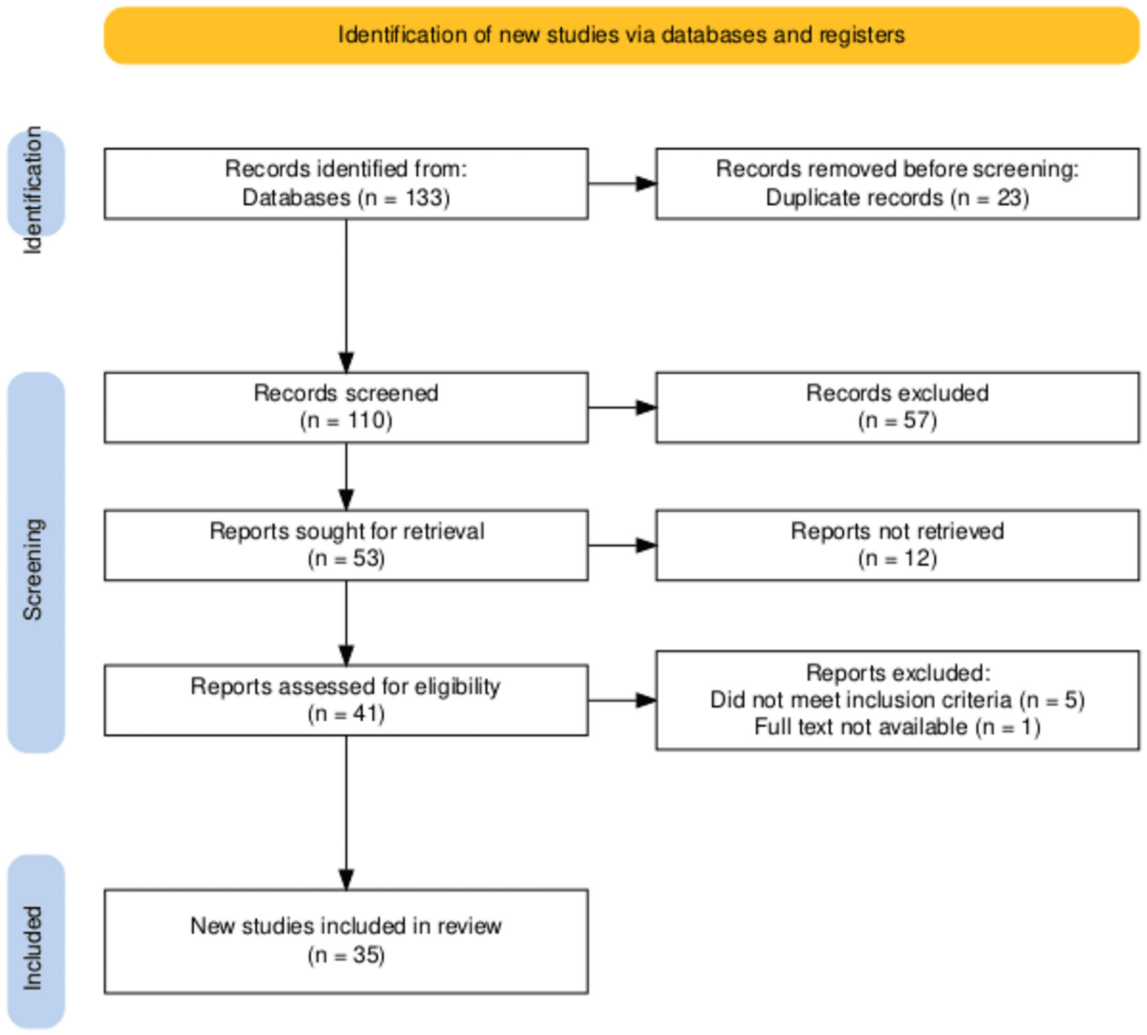
PRISMA 2020 flow diagram of study selection PRISMA: Preferred reporting items for systematic reviews and meta-analyses.

The initial database search yielded 133 records. After removing duplicates, 110 remained. Then, after performing initial title screening, 53 records remained. Abstract screening eliminated a further 12 articles, leaving 41 for full eligibility review. After applying inclusion and exclusion criteria, 36 articles met eligibility requirements, of which 35 had full texts available and were included in the final synthesis. The complete selection process is illustrated in Figure 1 (PRISMA flow diagram) [7].

Results

A total of 35 full-text articles were included in the final review, meeting predefined criteria [1-35]. The 35 included studies comprise systematic reviews, meta-analyses, population based longitudinal studies, cross- sectional analyses, hemodialysis cohort studies, and one pilot RCT. Most literature was published between 2015 and 2025, while select foundational studies from 1991 to 2014 were retained for mechanistic context.

Table 1 summarizes the key characteristics and findings of all included studies, organized by evidence hierarchy.

**Table 1:** Key characteristics and findings of included studies, organized by evidence hierarchy References denoted as "Foundation" were published before 2015 and retained for mechanistic context only. "Primary" denotes studies published 2015-2025 that form the core evidentiary base. AGE, advanced glycation end-products; CKD, chronic kidney disease; CRP, C-reactive protein; DM, diabetes mellitus; DMFT, Decayed Missing and Filled Teeth; ESRD, end-stage renal disease; GFR, glomerular filtration rate; Hgb, hemoglobin; KAPD, Kidney and Periodontal Disease trial; KDOQI, Kidney Disease Outcomes Quality Initiative; NHANES, National Health and Nutrition Examination Survey; NSPT, non-surgical periodontal treatment; PD, periodontal disease; PPD, periodontal pocket depth; RCT, randomized controlled trial.

Study author (year)	Type of study	Patient profile	Key outcomes measured/Results	Parameter studied	Study included as
Hajishengallis (2015) [1]	Review article (mechanistic foundation)	Periodontal disease – microbial and immune mechanisms	Described how periodontal pathogens subvert host immunity and drive systemic inflammation via NF-κB and ROS, reducing GFR	NF-κB, ROS, GFR	Foundation
Levey et al. (2003) [2]	Clinical practice guideline	General CKD population	Defined CKD as GFR <60 mL/min/1.73 m² or structural kidney damage >3 months; foundational CKD classification	GFR, albuminuria, serum creatinine	Foundation
Deschamps-Lenhardt et al. (2019) [4]	Systematic review & meta-analysis	CKD patients vs. periodontally healthy controls	Moderate-to-severe PD associated with 2.5-fold higher prevalence of CKD and nearly double the risk of incident renal decline	GFR, serum creatinine, periodontal indices	Primary
Galli et al. (2025) [5]	Systematic review & meta-analysis	CKD and ESRD patients	Higher prevalence and severity of PD with declining renal function; support integrated dental-nephrological care	GFR, periodontal indices, CRP	Primary
Ioannidou et al. (2014) [6]	Cross-sectional study	CKD patients (NHANES cohort)	Tooth loss independently associated with malnutrition markers in CKD; elevated DMFT scores	DMFT, serum albumin, BMI, malnutrition indices	Foundation
Bullón et al. (2025) [8]	Review article	Periodontal disease – mechanistic focus	Detailed role of oxidative stress in periodontitis; ROS as shared mediator of periodontal and renal endothelial injury	ROS, oxidative stress markers, GFR	Primary
Delamaire et al. (1997) [10]	Clinical study	Patients with diabetes mellitus	Demonstrated hyperglycemia impairs neutrophil chemotaxis and phagocytosis	Neutrophil function assays, HbA1c	Foundation
Chang et al. (2017) [16]	Population-based longitudinal study	General adult population (Taiwan)	Deeper periodontal pockets independently associated with CKD progression; hyperglycemia accelerated renal deterioration	GFR, HbA1c, PPD	Primary
Siribamrungwong et al. (2012) [18]	Interventional study	Maintenance hemodialysis patients	Periodontal treatment significantly reduced systemic inflammatory markers	CRP, albumin, Kt/V	Foundation
Fisher et al. (2008) [20]	Cross-sectional study	General adult population	PD identified as independent, nontraditional risk factor for CKD	GFR, serum creatinine, periodontal indices	Foundation
Rapone et al. (2019) [21]	Observational cohort study	Hemodialysis patients	Periodontal inflammation correlated with nutritional deficits and systemic inflammatory burden	CRP, albumin, malnutrition-inflam mation score	Foundation
Naghsh et al. (2017) [22]	Cross-sectional study	Hemodialysis patients	PD associated with altered serum biochemistr, including calcium, phosphate, albumin, and creatinine	Albumin, creatinine, calcium, phosphate, CRP	Foundation
Cotič et al. (2017) [23]	Cross-sectional study	Hemodialysis patients	Plaque index correlated with albumin, calcium, phosphate, ferritin, and hemoglobin	Albumin, calcium, phosphate, ferritin, Hgb, plaque index	Foundation
Sharma et al. (2016) [25]	NHANES linked mortality study	CKD stages 3–5	PD associated with all-cause and cardiovascular mortality in CKD	Mortality records, GFR, cardiovascular events	Foundation
Ricardo et al. (2015) [26]	NHANES linked mortality study	CKD patients	Confirmed association between PD and mortality in CKD; effect attenuated after adjustment	Mortality records, GFR, CRP, albumin	Foundation
Delbove et al. (2021) [27]	Systematic review	CKD patients with PD	Evaluated the effect of periodontal treatment on GFR and systemic inflammatory markers	GFR, CRP, albumin	Primary
Silva et al. (2024) [29]	Cross-sectional clinical assessment	CKD patients (Saudi cohort)	Characterized oral health challenges, including xerostomia, uremic stomatitis, and bleeding risk	Clinical oral findings, bleeding indices, xerostomia scores	Primary
Mouradian et al. (2014) [30]	Cross-sectional policy review	Dentistry-medici ne integration articles	Summarized drivers of dental-medical integration and interprofessional collaboration	Collaboration metrics, quality outcomes	Foundation
Grubbs et al. (2019) [31]	Pilot RCT (KAPD trial)	CKD patients with co-existing PD	NSPT showed limited evidence of benefit on renal function; established feasibility for future RCTs	GFR, CRP, HbA1c, periodontal pocket depth	Primary
Eburne et al. (2025) [32]	Systematic review	CKD and periodontitis populations	Updated epidemiology and biological mechanisms linking periodontitis with CKD; updated mortality hazard ratios	Mortality HR, GFR, periodontal indices	Primary
Jeffcoat et al. (2014) [33]	Retrospective insurance data study	Patients with systemic conditions, including CKD	Periodontal therapy associated with reduced medical costs and improved outcomes	Claims data, hospitalization rates, systemic disease event rates	Foundation
Li et al. (2025) [34]	Observational review	Patients with PD and CKD	Reviewed epidemiological and pathogenic mechanisms; explored CKD prevention through PD intervention	Pathogenic mechanisms, therapeutic targets	Primary
Mottl et al. [35]	KDOQI commentary on the KDIGO 2022	Diabetes management in CKD.	Bidirectional relationship between periodontitis and CKD	Periodontal care may represent an important and novel management strategy for individuals with CKD.	Primary
Gudsoorkar et al. (2025) [36]	Policy perspective / narrative review	People with CKD, especially DM and transplant candidates	Periodontal therapy may lower HbA1c, reduce inflammation, and potentially slow CKD progression; proposes integrated care systems	HbA1c, integrated care outcomes	Primary

Discussion

Proposed Biological Mechanisms of the PD-CKD Axis

**Figure 2:**
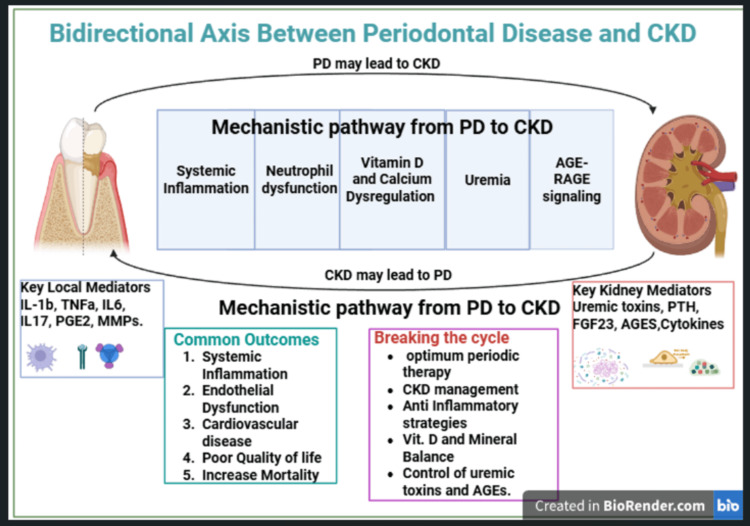
Conceptual schematic presentation of the bidirectional PD-CKD axis The figure incorporates the NF-κB/ROS pathways, neutrophil dysfunction, vitamin D dysregulation, uremia, AGE-RAGE signaling, and collagen metabolism disruption. NF-κB, Nuclear factor kappa-light-chain-enhancer of activated B cells; ROS, Reactive oxygen species; AGEs, advanced glycation end-products; AGE-RAGE, advanced glycation end product receptor for advanced glycation end products; CKD, chronic kidney disease; FGF23, fibroblast growth factor 23; IL-1β, interleukin-1 beta; IL-6, interleukin-6; IL-17, interleukin-17; MMPs, matrix metalloproteinases; PD, periodontal disease; PGE2, prostaglandin E2; PTH, parathyroid hormone; RAGE, receptor for advanced glycation end-products; TNF-α, tumor necrosis factor-alpha. Image credit: Created by authors using BioRender.com (BioRender Inc., Toronto, ON, Canada).

Figure 2 outlines the key mechanisms in the PD-CKD axis.

Vitamin D metabolism and calcium homeostasis: Diabetes mellitus is a leading cause of CKD; progressive renal impairment disrupts vitamin D metabolism, thereby impairing calcium absorption. This disruption triggers secondary hyperparathyroidism, which mobilizes calcium from osseous stores, including the alveolar bone. Vitamin D deficiency also attenuates anti-inflammatory responses, exacerbating periodontal inflammation. Altered calcium and phosphorus homeostasis in CKD may reduce alveolar bone mineral density; however, clinically observed tooth mobility in CKD patients is primarily attributable to underlying periodontal bone loss rather than systemic calcium depletion alone. Enamel hypoplasia has been documented in patients with early-onset or pediatric CKD history [6].

The primary proposed mechanism linking these conditions is inflammatory mediation. Periodontal pathogens can enter the bloodstream through ulcerated epithelium, causing transient bacteremia or releasing immunostimulatory byproducts that activate the innate immune system. This activates the NF-κB pathway and increases the production of reactive oxygen species (ROS), leading to dysregulated systemic inflammation that damages renal endothelial cells and reduces the GFR [1,8,9].

Impaired innate immune function (neutrophil dysfunction): Neutrophils constitute the primary innate defense against the dysbiotic biofilm responsible for gingivitis and periodontitis. Hyperglycemia impairs neutrophil chemotaxis and phagocytosis, reducing bacterial clearance at periodontal sites. This impaired innate immune function allows periodontal pathogens to proliferate and invade deeper periodontal structures, accelerating disease progression [10,11].

Uremia and the oral environment: CKD leads to uremia, defined by the accumulation of uremic toxins. In the oral cavity, elevated urea is converted to ammonia, causing xerostomia, metallic dysgeusia, and reduced appetite. This environment often leads to malnutrition, which further impairs immune responses and worsens periodontal status. Platelet dysfunction in uremia may contribute to unresolved gingival bleeding, increasing infection risk, and predisposing to PD [5,12].

Advanced glycation end-products (AGEs): Under sustained hyperglycemia, glucose molecules permanently bind to proteins and lipids via a non-enzymatic process, forming advanced glycation end-products (AGEs). AGEs accumulate within gingival tissues and bind to their cognate receptor (RAGE), initiating a cascade of pro-inflammatory cytokine release. This hyperinflammatory state accelerates destruction of the alveolar bone and periodontal connective tissue [12-14].

Altered collagen metabolism and tissue repair: A healthy periodontium depends on a balanced anabolic and catabolic phase of collagen turnover. Hyperglycemia shifts this balance by stimulating overproduction of matrix metalloproteinases (MMPs), which degrade collagen, while simultaneously suppressing fibroblast activity required for collagen synthesis. This metabolic deficit accelerates the formation of deep periodontal pockets, loss of clinical attachment, and eventual tooth mobility [15,16].

Hyperglycemic oral environment and dysbiotic microbial risk: In poorly controlled DM, elevated glucose levels in gingival crevicular fluid provide a nutrient source for periodontal pathogens, shifting the oral microbiome toward a more aggressive dysbiotic community and increasing both the risk and severity of periodontal infection [17-19]. Physicians prescribing GLP-1 receptor agonists should counsel patients about xerostomia as a potential adverse effect that may serve as a nidus for dysbiotic pathogen proliferation and encourage regular dental review.

Clinical Biomarkers and Disease Monitoring

Dental scales: CKD patients have significantly elevated Decayed, Missing, and Filled Teeth (DMFT) scores and greater tooth loss compared to healthy controls [20].

Apical periodontitis and renal function: Studies report that 75.2% of CKD patients had at least one tooth with apical periodontitis (AP), compared to 40.9% in controls; severity of AP also correlates with increased serum creatinine and decreased GFR [21].

Periodontal pocket depth and CKD progression: Population-based longitudinal data demonstrate that deeper periodontal pockets are significantly associated with renal function decline, with hyperglycemia acting as a potent synergistic mediator of both periodontal and renal deterioration [10,22].

Serum biomarkers: Periodontitis in hemodialysis patients is associated with lower serum albumin and phosphate levels and higher C-reactive protein (CRP) [22]. Cotič et al. further identified a significant correlation between the visible plaque index and serum albumin, calcium, phosphate, ferritin, and hemoglobin, suggesting that oral inflammation severity reflects systemic metabolic and nutritional status [23].

Management Challenges and Pharmacological Cautions

GLP-1 receptor agonists and xerostomia: GLP-1 receptor agonists reduce hyperglycemia, which may, in turn, reduce the risk of PD. However, xerostomia associated with these agents may increase the risk of dysbiotic pathogen proliferation at periodontal sites. Clinicians prescribing GLP-1 agonists for DM management or weight reduction should advise patients to maintain strict dental hygiene and to schedule regular dental evaluations for monitoring dry mouth [24,25].

Cardiovascular and all-cause mortality risk: The association between periodontitis and mortality in the CKD population remains an active area of investigation. Sharma et al. (2016) reported an association between PD and all-cause and cardiovascular mortality in CKD stages 3-5; however, this association was heterogeneous across pooled studies, and causality has not been established [25]. Ricardo et al. and Delbove et al. further demonstrated that the mortality association was attenuated after comprehensive comorbidity adjustment, highlighting the substantial confounding by shared risk factors [26,27].

Pharmacological cautions for nephrotoxic medications: Early pharmacological management with awareness of nephrotoxic medications is essential in CKD patients requiring periodontal treatment. Nephrotoxic antibiotics (e.g., aminoglycosides) and non-steroidal anti-inflammatory drugs must be avoided given their potential to reduce GFR and exacerbate renal impairment [28-30].

Non-surgical periodontal treatment and renal outcomes: While lifestyle modifications, including smoking cessation and optimized oral hygiene, are universally recommended, NSPT currently lacks sufficient evidence of significant improvement in renal function. The KAPD pilot RCT (Grubbs et al., 2019) [31] and a 2021 systematic review (Delbove et al.) [27], both highlight the need for larger, adequately powered RCTs before clinical recommendations can be made.

Integrated Care and Collaborative Practice

The shift toward medical-dental integration is supported by major professional organizations, with evidence from insurance data demonstrating that periodontal therapy is associated with reduced hospitalizations and medical costs across multiple systemic conditions [33,34]. 

For patients with CKD stage two or higher, increased follow-up frequency (every two to three months) is recommended to mitigate the rapid progression of metabolic bone disease. Collaborative dental medical integration, including common grand rounds, interdisciplinary case review, and communication between dental and medical practitioners, holds significant promise for improving the management of patients with co-existing PD and CKD [35]. Gudsoorkar et al. (2025) propose a system-level framework in which integrated care could yield better oral screening, improved care coordination, reduced inequities, fewer hospitalizations, and lower long-term healthcare costs [36].

Research Gaps and Future Directions

Despite strong associative data, a significant knowledge gap persists regarding the definitive causal architecture of the PD-CKD axis. Current literature is frequently confounded by smoking, socioeconomic status, and shared metabolic comorbidities, obscuring whether the relationship is truly bidirectional, unidirectional, or a manifestation of a shared systemic inflammatory "third factor" [26].

The prognostic value of PD in predicting all cause and cardiovascular mortality within the CKD population remains a point of clinical controversy, primarily due to high methodological heterogeneity in pooled studies and a scarcity of high powered longitudinal data targeting end-stage renal disease cohorts [31].

Future research must prioritize standardized longitudinal studies utilizing uniform periodontal diagnostic criteria and renal biomarkers. Large scale RCTs should stratify outcomes by CKD secondary to DM and CKD due to non-diabetic etiologies (e.g., hypertension or glomerulonephritis) to disentangle the specific role of hyperglycemia mediated inflammation from other pathways of renal and periodontal destruction.

Limitations

This review has several methodological limitations that should be acknowledged. First, this manuscript was not pre-registered on PROSPERO or any equivalent systematic review registry, which limits the full reproducibility of the study selection process. Second, given the significant heterogeneity in study designs, periodontal case definitions, patient populations, and renal biomarker reporting, a formal meta-analytic pooling of effect estimates was not feasible, and findings are synthesized narratively. Third, while a risk-of-bias assessment using a validated tool (e.g., Newcastle-Ottawa Scale, Grading of Recommendations Assessment, Development, and Evaluation (GRADE), or A Measurement Tool to Assess systematic Reviews (AMSTAR)) was not performed, the evidence hierarchy presented in Table 1 provides transparency regarding the relative methodological strength of included studies. Fourth, select studies published prior to 2020 were retained for mechanistic context; their inclusion reflects pragmatic disclosure rather than strict adherence to the stated 2020-2025 primary search window. Prospective systematic reviews on this topic should incorporate PROSPERO registration, dual reviewer data extraction, and formal quality appraisal.

## Conclusions

Current evidence underscores a bidirectional association between compromised periodontal health and the progression of CKD, primarily driven by shared inflammatory mediators, oxidative stress, and metabolic dysregulation. Critically, the available literature conflates CKD-driven mechanisms, such as uremia, vitamin D dysregulation, immune suppression, and altered calcium homeostasis, with pathways specific to DM-associated CKD, including AGE accumulation and neutrophil impairment secondary to hyperglycemia. Future research must stratify CKD etiologies to delineate these pathways clearly.

A significant evidentiary gap persists due to a lack of standardized diagnostic criteria across longitudinal studies, making it difficult to definitively determine whether PD serves as an independent contributing factor to renal decline, distinct from the established primary etiologies of CKD, such as uncontrolled DM and hypertension, or is primarily a secondary manifestation of systemic uremia. Until prospective trials adopt uniform periodontal and renal biomarkers and are adequately powered to stratify by CKD etiology, the PD-CKD axis will remain partially obscured.

Given this complexity, treating the oral cavity and renal system in isolation is no longer clinically sufficient. An integrated care model, involving seamless coordination between dental and medical providers, early pharmacological management with awareness of nephrotoxic medications, and the incorporation of dental screenings into standard renal care protocols, is essential for improving the management of patients with co-existing PD and CKD.
